# 14-3-3ζ loss leads to neonatal lethality by microRNA-126 downregulation-mediated developmental defects in lung vasculature

**DOI:** 10.1186/s13578-017-0186-y

**Published:** 2017-11-02

**Authors:** Jun Yang, Sonali Joshi, Qingfei Wang, Ping Li, Hai Wang, Yan Xiong, Yi Xiao, Jinyang Wang, Jan Parker-Thornburg, Richard R. Behringer, Dihua Yu

**Affiliations:** 1 Department of Molecular and Cellular Oncology, Unit 108, The University of Texas MD Anderson Cancer Center, 1515 Holcombe Boulevard, Houston, TX 77030 USA; 2 Department of Genetics, The University of Texas MD Anderson Cancer Center, Houston, TX 77030 USA; 3 University of Texas Health Science Center Graduate School of Biomedical Sciences, Cancer Biology Program, Houston, TX 77030 USA; 4 Center for Molecular Medicine, China Medical University, Taichung, 40402 Taiwan

**Keywords:** 14-3-3ζ, miR-126, Lung development, Angiogenesis

## Abstract

**Background:**

The 14-3-3 family of proteins have been reported to play an important role in development in various mouse models, but the context specific developmental functions of 14-3-3ζ remain to be determined. In this study, we identified a context specific developmental function of 14-3-3ζ.

**Results:**

Targeted deletion of 14-3-3ζ in the C57Bl/6J murine genetic background led to neonatal lethality due to respiratory distress and could be rescued by out-breeding to the CD-1 or backcrossing to the FVB/NJ congenic background. Histological analysis of lung sections from 18.5 days post coitum embryos (dpc) showed that 14-3-3ζ−/− lung development is arrested at the pseudoglandular stage and exhibits vascular defects. The expression of miR-126, an endothelial-specific miRNA known to regulate lung vascular integrity was down-regulated in the lungs of the 14-3-3ζ−/− embryos in the C57Bl/6J background as compared to their wild-type counterparts. Loss of 14-3-3ζ in endothelial cells inhibited the angiogenic capability of the endothelial cells as determined by both trans-well migration assays and tube formation assays and these defects could be rescued by re-expressing miR-126. Mechanistically, loss of 14-3-3ζ led to reduced Erk1/2 phosphorylation resulting in attenuated binding of the transcription factor Ets2 on the miR-126 promoter which ultimately reduced expression of miR-126.

**Conclusion:**

Our data demonstrates that miR-126 is an important angiogenesis regulator that functions downstream of 14-3-3ζ and downregulation of miR-126 plays a critical role in 14-3-3ζ-loss induced defects in lung vasculature in the C57Bl/6J genetic background.

**Electronic supplementary material:**

The online version of this article (10.1186/s13578-017-0186-y) contains supplementary material, which is available to authorized users.

## Background

The mammalian 14-3-3 protein family consists of seven isoforms (β, τ, ε, ζ, η, γ and σ). They modulate multiple signaling pathways in diverse biological processes by binding to specific phospho-specific motifs on target proteins and thereby regulate their function, activity, stability, or localization [1, 2]. Due to their ability to interact with a plethora of target proteins, they are reported to play an important role in the regulation of cell cycle, DNA damage repair, cell proliferation, cell polarity, programmed cell death and cell metabolism, etc. [3–8]. In spite of exhibiting a high degree of homology, knockout mouse models of different 14-3-3 family members have revealed distinctive biological functions for the different isoforms [1, 9, 10], indicating that the various isoforms can exert unique functions which cannot be compensated by other members of the 14-3-3 family.

14-3-3ζ has been implicated in various human diseases such as Alzheimer’s disease [11, 12], progressive multifocal leukoencephalopathy [13], Huntington disease [14], Creutzfeldt–Jakob disease [15] and is reported to be upregulated in multiple cancer types, including lung [16, 17], breast [2, 18, 19], ovarian [20], head and neck [21] as well as lymphomas [22].

Previous studies on the developmental role of 14-3-3ζ have identified that 14-3-3ζ functions in neuronal development and in regulation of adipogenesis [23]. On a SV129 background, 14-3-3ζ−/− mice were reported to exhibit behavioral and cognitive defects similar to those observed in schizophrenia, autism spectrum disorder and bipolar disorder [24]. Additionally, these mice were found to manifest enhanced locomotor hyperactivity due to dysregulation of dopamine signaling [25]. On a Balb/c background, deletion of 14-3-3ζ was found to result in hippocampal defects with a decrease in spatial memory but they did not exhibit enhanced locomotor activity as observed on a SV129 background [26]. 14-3-3ζ−/− mice have also been reported to exhibit reduced deposition of visceral fat as loss of 14-3-3ζ promotes autophagy mediated degradation of CEBP/δ preventing induction of the master adipogenesis regulators PPARγ and C/EBPα [23]. However, these previous studies did not reveal a role of 14-3-3ζ in lung development.

In this study, we generated a 14-3-3ζ hypomorphic mutant mouse (14-3-3ζ−/−) model for studying the 14-3-3ζ gene function in vivo. We found that targeted deletion of 14-3-3ζ on a C57Bl/6J genetic background led to neonatal lethality due to respiratory distress, which resulted from miR-126 downregulation-induced defects in lung vasculature. Our findings demonstrate a unique and essential role of 14-3-3ζ in normal development in a genetic background specific manner.

## Methods

### Generation and maintenance of 14-3-3ζ knockout mouse

The ES cell line RRR334, in which 14-3-3ζ was targeted according to the 5′RACE data from the Baygenomics database, was obtained from Mutant Mouse Regional Resource Center (MMRRC). RT-PCR was performed to confirm that the cell line inactivates 14-3-3ζ. The primer sequence for the exogenous was forward: 5′-TGCTGAGAAAAAGCAGCAGA and reverse: 5′-GACAGTATCGGCCTCAGGAAGATCG. The primer sequence for endogenous 14-3-3ζ control PCR was forward: 5′-TGCTGAGAAAAAGCAGCAGA and reverse: 5′-TTGTCATCACCAGCAGCAAC. The genetic engineered mouse facility at MD Anderson Cancer Center injected the cells into B6(Cg)-Tyr^c − 2j^/J (The Jackson Laboratory, Bar Harbour, ME, Stock #000058) blastocysts. Chimeras were mated with B6(Cg)-Tyr^c − 2j^/J mice to test germline transmission and obtain 14-3-3ζ knockout founder mice. The primer sequences for PCR genotyping were forward: 5′-CAACCATGTTGGGATAGAGG homologous to 14-3-3ζ intron 3, reverse: 5′-CCAAATAAGCCTTCCCTTCC homologous to intron 3 and 5′-AAGGGTCTTTGAGCACCAGA homologous to the gene trap vector. PCR resulted in 954-bp fragment from the wild-type allele and a 544-bp fragment from the mutant allele. Mice were backcrossed into C57Bl/6J and FVB/NJ congenic background as determined by genome scan using a panel of simple sequence length polymorphism (SSLP) (microsatellite) markers, or outbred to CD-1 genetic background respectively and maintained thereafter. The C57Bl/6J and FVB/NJ mouse breeders were purchased from the Jackson Laboratory (Bar Harbor, ME). The CD-1 breeders were purchased from Charles Rivers (Wilmington, MA). All animal work was performed under an IACUC-approved protocol. University of Texas MD Anderson Cancer Center is an AAALAC accredited institution.

### Cell culture

The mouse endothelial cell line ARBEC was obtained from Dr. Fidler’s lab at MDACC. The endothelial cells were generated from H-2Kb-tsA58 mice immortalized with SV40 large T antigen. The ARBEC cells were cultured using DMEM supplemented with 10% FBS. The transfection was performed using Lipofectamine 2000 using standard protocol.

MCF7 cells were cultured using DMEM supplemented with 10% FBS. N-terminal 139aa of 14-3-3ζ was cloned into pcDNA3 and later transfected into MCF7 cell lines. Stable clones (∆C1 and ∆C12) were selected with G418 and high expressers were examined by western blot and maintained since. Mouse embryonic fibroblast (MEF) cells were obtained from E13.5 B6 embryos. The embryos were minced, cut and filtered. Cells were then centrifuged, resuspended in DMEM supplemented with 10% FBS and plated. Control pre-microRNA and pre-miR126 were ordered from Exiqon.

### Tissue collection and histological analysis

The mouse embryos were collected by C-section at 18.5 dpc (days post coitum). The lung tissues were collected after euthanasia following an IACUC-approved protocol. Tissues were fixed in 10% neutral buffered formalin for 12–18 h. The samples were stored in 70% ethanol and then embedded in paraffin. Paraffin sections (5 µm) were stained with hematoxylin and eosin. Histological analysis were independently evaluated by at least two pathologists (Y.X., W.H. and Q.Z.). Immunohistochemistry (IHC) was performed as previously described [19]. Antibodies used were Ki67 (DAKO, Carpentaria, CA M7249), CD34 (eBioscience, San Diego, CA 14-0341), 14-3-3ζ (C-16, Santa Cruz, Santa Cruz, CA sc-1019), SP-A (Santa Cruz sc-13977), and AQP5 (Calbiochem, Germany 178615). For IHC analysis and quantification, 10 fields were randomly chosen at 200 × magnification. The total number of cells and positive cells were counted, and the average percentage of positive cells was determined.

### Immunoblotting

Tissues were collected from the mice. Protein extracts were prepared by homogenizing samples in tissue lysis PBSTDS buffer [10 mmol/L sodium phosphate (pH 7.3), 154 mmol/L NaCl, 5% sodium deoxycholate, 1% SDS] using a tissue grinder, followed by centrifugation to remove particulate matter and lipids. Immunoblotting was performed as previously described by Lu et al. [19]. Antibodies used were anti-HA high affinity (clone 3F10, Roche 11867423001), 14-3-3ζ (C-16, Santa Cruz sc-1019), Erk (Cell Signaling 4695), phosphor-Erk (T202/Y204, Cell Signaling 4370), Mek1 (Cell Signaling 9126), phosphor-Mek1 (S221, Cell Signaling 2338), Ets1 (c-20, Santa Cruz sc-350), Ets2 (c-20, Santa Crux sc-351), Akt (Cell Signaling 9272), phosphor-Akt (S473, Cell Signaling 3787), β-actin (Sigma A5441), and tubulin (Sigma T5168). The antibodies were mainly used at a 1:1000 dilution.

### Quantitative RT-PCR for microRNA quantification

RNA of the tissue samples was extracted using Trizol (Invitrogen, Carlsbad, CA) following the manufacturer’s instructions. The specific primers and probe for miR-126 real-time PCR were purchased from Applied Biosystems. Reverse transcription was performed using the Taqman MicroRNA Reverse Transcription kit (Applied Biosystems 4366569), and real-time PCR was performed using the Taqman Universal PCR Master Kit (Applied Biosystems 4324018) and iQ SyBR Green Supermix (Bio-Rad 170-8882). The primer sequences for PECAM1 were forward: 5′-CTGGTGCTCTATGCAAGCCT and reverse: 5′-AGTTGCTGCCCATTCATCAC. The primer sequences for 18S ribosomal RNA were forward: 5′-AACCCGTTGAACCCCATT and reverse: 5′-CCATCCAATCGGTAGTAGCG. Control pre-microRNA and pre-miR126 were ordered from Exiqon.

### Transwell migration assay

Mouse endothelial cells (3 × 10^4^) resuspended in 500 µL of serum-free medium were seeded in the top chamber of 24-well 8-µm pore transwell plates. 600 µL of medium containing 10% FBS was used as chemo-attractant in the bottom chamber. The plates were incubated at 37 °C for 4 h. The cells were fixed in 10% neutral buffered formalin for 1 h before staining with 0.5% crystal violet for 1 h. The plates were then flushed under tap water to remove excessive dye. The cells that did not migrate through the wells were wiped away by cotton tips. Images were taken with a Zeiss Discovery V20 microscope with the supplied software Axiom. The entire wells were quantified and three replicates were performed for statistical analysis.

Quantification of the migrated cells was performed using Adobe Photoshop and ImageJ software.

### Tube formation assay

Matrigel (BD, Franklin Lakes, NJ 356231) was thawed at 4 °C overnight before assay, and then 500 µL of Matrigel was added into each well of the 24-well plate. The plates were incubated at 37 °C for 30 min to 1 h to form gel. Cells (3 × 10^5^) resuspended in DMEM/F-12 medium containing 10% FBS were plated above the Matrigel and incubated for 6 h at 37 °C. The formation of the tube structure by the endothelial cells was imaged with an Olympus inverted fluorescence microscope and quantified using the ImageJ software. Three replicates were performed and five images were taken from each well for analysis.

### Statistical analyses

Statistical differences were assessed with two-tailed Student’s t test or one-way ANOVA. A *p* value of < 0.05 was considered statistically significant.

### ChIP assay

Chromatin proteins were cross linked to chromatin with formaldehyde and sheared into 400–1000 bp fragments. Nucleoprotein complexes were immunoprecipitated using antibody to Ets-1, Ets-2, or control IgG antibody. The precipitated DNA fractions were analyzed by Quantitative-PCR for the presence of the miR-126 proximal regulatory region encompassing the EBS1 and EBS2 (region − 150 to + 100 bp). Input DNA was used as a positive control.

## Results

### Generation of 14-3-3ζ knockout mice

**Fig. 1:**
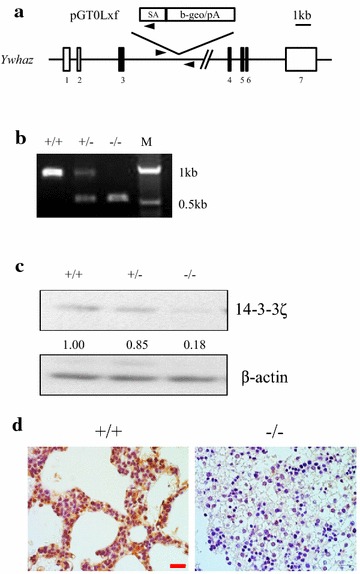
Generation and characterization of 14-3-3ζ knockout mice. **a** Schematic map showing that the gene trap vector integrated into intron 3 of the 14-3-3ζ gene (Ywhaz). Ywhaz is the HUGO Gene Nomenclature Committee—approved gene symbol for 14-3-3ζ. The lines represent the introns for the 14-3-3ζ gene and the rectangles signify the exons. The solid sections indicate the coding region for 14-3-3ζ protein. The gene trap vector pGT0Lxf was integrated ~ 3.3 kb downstream of exon 3. SA indicates the splice acceptor sequence of mouse En2 exon 2. β-Geo/pA indicates the fusion of β-galactosidase and neomycin transferase followed by SV40 polyadenylation signal. The arrows indicate the primers for genotyping. The scale bar represents 1 kb length of DNA sequence. **b** PCR genotyping for 14-3-3ζ knockout mice: +/+, +/-, and −/− indicate 14-3-3ζ wild-type, heterozygous, and homozygous mutant alleles, respectively. M indicates marker. Wild-type allele generated a 954-bp band, and the mutant allele generated a 544-bp PCR product. **c** Western blot confirming the loss of 14-3-3ζ expression in mouse embryonic fibroblast (MEF) cells. β-Actin served as loading control. Quantification of relative 14-3-3ζ expression level was shown below the western panel. **d** Immunohistochemical (IHC) staining of 14-3-3ζ expression in the neonatal lung tissue dissected from +/+ and −/− mice. Scale bar indicates 50 µm in length

A gene trap approach was used to generate 14-3-3ζ knockout mice. RRR334 ES cells were obtained from the Mutant Mouse Regional Resource Center (MMRRC) and the disruption of 14-3-3ζ at exon 3 was confirmed by RT-PCR (Additional file 1: Figure S1A). The integration site of the gene trap vector was found to be located 3351 bp downstream of exon 3 in the 14-3-3ζ gene by PCR amplification followed by DNA sequencing (Additional file 1: Figure S1B). The site of genomic integration was further confirmed by southern blotting (data not shown). Based on the integration site, a forward primer homologous to intron 3 and a reverse primer homologous to the gene trap vector were used for genotyping (Fig. 1a, b). The ES cells were injected into C57Bl/6J albino mouse blastocysts and chimera mice with high ES cell contribution based on their fur color were mated with C57Bl/6J albino mice to obtain 14-3-3ζ heterozygous (+/-) founder mice. The founder male and female 14-3-3ζ+/- mice were mated to generate 14-3-3ζ homozygous mutant (−/−) mice. 14-3-3ζ protein expression was significantly reduced or lost in MEF cells as well as multiple tissue types from the knockout mouse (Fig. 1c, d and Additional file 1: Figure S2C).

Based on the integration site of the gene trap vector, the knockout mouse should express a truncated 14-3-3ζ protein containing 139 amino acids of the N-terminal part of 14-3-3ζ protein fused to the β-geo cassette. To test whether the N-terminal part of 14-3-3ζ protein is functional, a vector containing the HA-tagged N-terminal fragment of 14-3-3ζ with deletion of the C-terminal target protein binding groove (ΔC14-3-3ζ) [27, 28] was transfected into MCF7 breast cancer cells. The expression of the HA-tagged ∆C14-3-3ζ was detectable only in the presence of the proteosomal inhibitor MG132 (Additional file 1: Figure S2A) and it did not enhance cell proliferation or induce activation of the Ras/Raf/Erk and PI3K/Akt pathways (Additional file 1: Figure S2B, C) as would be expected with full length 14-3-3ζ [29]. Thus the N-terminal fragment of 14-3-3ζ is non-functional and the 14-3-3ζ−/− mouse generated by the gene-trap approach is a strong hypomorphic model for studying the function of 14-3-3ζ in vivo.

### 14-3-3ζ−/− mice are neonatal lethal with defective lung development

After mating heterozygous male and female founders, approximately 5% of the litter was homozygous for the 14-3-3ζ gene trap allele, as determined by genotyping 10 days after birth (Table 1). Failure to yield the expected 25% homozygous births suggested that 14-3-3ζ-loss may be lethal either during embryogenesis or soon after birth [30, 31]. Genetic background can be an important determinant of phenotypes observed in knockout mice [32, 33]. We backcrossed the gene trap allele on to a congenic C57Bl/6J genetic background. 14-3-3ζ expression level was remarkably downregulated in this genetic background (Fig. 1c, d). Remarkably, genotyping revealed no surviving homozygous mutant pups (Table 1), confirming the lethal phenotype observed on the B6/129P2 F2 hybrid genetic background strain. To test whether out-crossing to different genetic backgrounds could circumvent the lethal phenotype, 14-3-3ζ heterozygous mice were bred on the CD-1 outbred strain. Heterozygous crosses resulted in ~ 25% homozygous mutant viable pups (Table 1 and Additional file 1: Figure S3A). Backcrossing the C57Bl/6J congenic 14-3-3ζ heterozygous mice to the FVB/NJ congenic background also resulted in viable pups at the expected ratios (Table 1 and Additional file 1: Figure S3B). The expression of other 14-3-3 isoforms was unaltered by loss of 14-3-3ζ (Additional file 1: Figure S3C). Thus, outbreeding to CD-1 and FVB/NJ background rescued the neonatal lethality observed on the C57Bl/6J strain.

**Table 1:** Genotype of pups generated by mating heterozygous breeders under different genetic background

Strain\geno	+/+	±	−/−	p value
B6/129P2 F2	22	39	3	0.0052
B6 congenic	58	72	0	< 0.0001
B6-E18.5	16	32	15	0.9879
CD-1	36	73	38	0.9849
FVB/NJ	27	53	25	0.9809

**Fig. 2:**
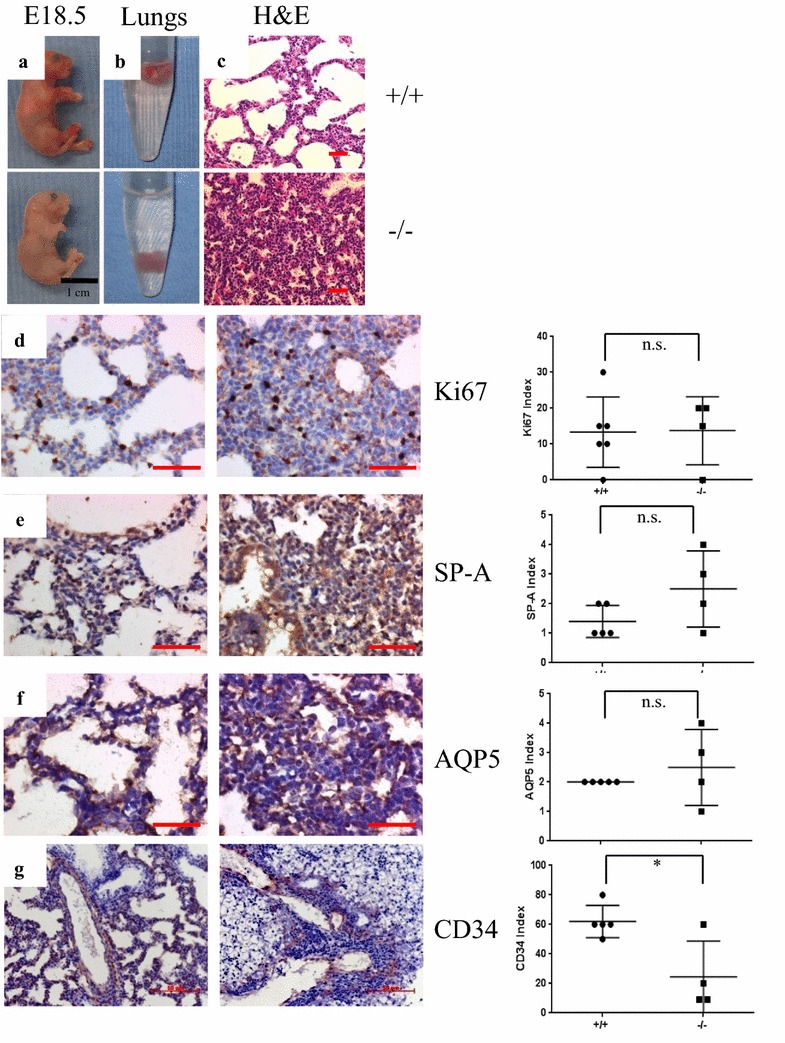
14-3-3ζ mediated neonatal lethality and associated defects in lung development. **a** 18.5 dpc embryos were dissected using caesarean section. Representative photos of 14-3-3ζ wild type (+/+) and homozygous mutant (−/−) are shown as indicated. **b** Lung tissues from the embryos were immersed in water to determine buoyancy. **c** Hematoxylin and eosin staining of the lungs dissected from the embryos. Sample size is indicated. **d** IHC staining for Ki-67 on the lungs dissected from +/+ and −/− embryos. **e** IHC staining for SP-A on the lungs dissected from +/+ and −/− embryos. **f** IHC staining for AQP5 on the lungs dissected from +/+ and −/− embryos. **g** IHC staining for CD34 on the lungs dissected from +/+ and −/− embryos. Length of the scale bar represents 50 µm in each panel. **d**–**g** Right: the quantification of the IHC stainings by immunoreactive score (IRS). ***, **, * Indicate p < 0.001, 0.01, and 0.05, respectively. n.s.: not significant

To determine the developmental stage when the 14-3-3ζ−/− mice on the C57Bl/6J background die, we dissected embryos from heterozygous crosses at 18.5 days post coitum (dpc). The ratio of the knockout embryos matched the predicted Mendelian ratios (Table 1). However, the 14-3-3ζ−/− embryos exhibited a pale color and died minutes after birth indicating respiratory failure (Fig. 2a). The lungs of the wild-type (+/+) mice floated on water, whereas the lungs of the 14-3-3ζ−/− embryos and newborn pups sank in water (Fig. 2b), indicating a failure to inflate. These results demonstrated that neonatal lethality of the 14-3-3ζ−/− mice is due to respiratory failure.

Hematoxylin and eosin (H&E) staining of the lung sections of the 18.5 dpc embryos showed that the lungs of the 14-3-3ζ−/− mice lacked saccular structures and exhibited increased mesenchymal compartments and thickened saccular septae (Fig. 2c). Amid that 14-3-3ζ expression level was significantly downregulated (Fig. 1d), no significant difference in pneumocyte proliferation was detected by Ki-67 immunohistochemical (IHC) staining (Fig. 2d). Pneumocyte differentiation was not altered by loss of 14-3-3ζ as evident by IHC staining of type II pneumocyte marker, pulmonary surfactant-associated protein A (SP-A), and type I pneumocyte marker, aquaporin isoform 5 (AQP5) (Fig. 2e, f). Loss of 14-3-3ζ resulted in fragmented blood vessels that were surrounded by leukocyte aggregates and also decreased endothelial cells as analyzed by CD34 staining (Fig. 2g). Thus defects in lung vasculature result in the respiratory distress observed in the 14-3-3ζ−/− mice.

### Loss of 14-3-3ζ impedes angiogenesis via downregulation of miR-126

**Fig. 3:**
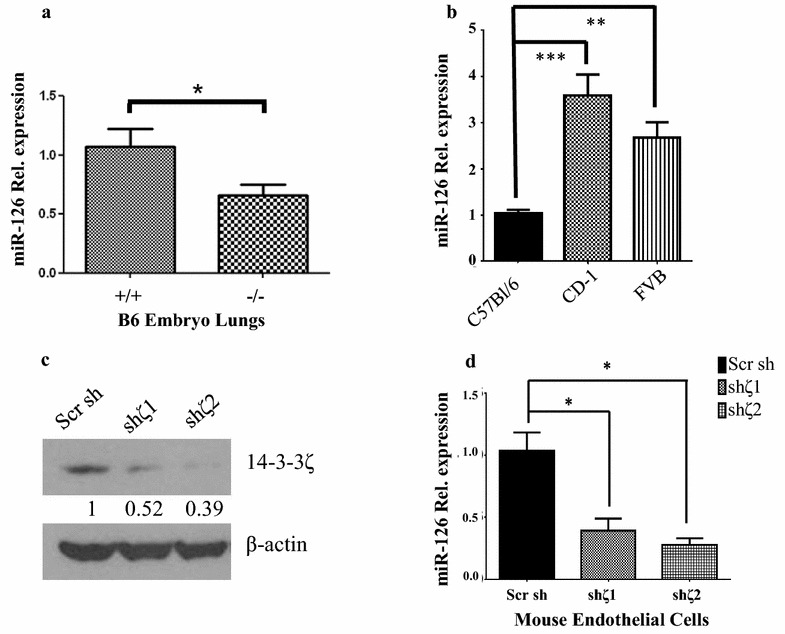
miR-126 regulates angiogenesis downstream of 14-3-3ζ. **a** qRT-PCR analysis of miR-126 expression normalized to PECAM1 in the lungs from 14-3-3ζ wild type (+/+) and knockout (−/−) embryo lungs, ± SD (n = 3) *p < 0.05. **b** qRT-PCR analysis of miR-126 expression normalized to PECAM1 in the lungs from 14-3-3ζ wild type (+/+) mice (6 weeks) from the indicated genetic backgrounds. **c** Detection of 14-3-3ζ and β-actin by western blotting in mouse endothelial cells expressing either a scrambled shRNA or shRNA targeting 14-3-3ζ. **d** qRT-PCR analysis of miR-126 expression normalized to PECAM1 in mouse endothelial cells expressing either a scrambled shRNA or shRNA targeting 14-3-3ζ. ± SD (n = 3)

Analysis of the Mouse Genome Informatics (MGI) database (http://www.informatics.jax.org/) revealed that targeted deletion of the endothelial specific miR-126 was reported to result in a lethal phenotype [34] similar to the 14-3-3ζ−/− mice on a C57Bl/6J genetic background. Quantitative RT-PCR analysis showed that miR-126 expression in the lungs of the 14-3-3ζ−/− embryos in the C57Bl/6J background was significantly reduced compared to their wild-type counterparts (Fig. 3a). Notably, miR-126 expression in the lungs of wild-type C57Bl/6J mice is significantly lower than that in the lungs of FVB/NJ and CD-1 mice of the same age (Fig. 3b), suggesting that further attenuating miR-126 expression in the already lower miR-126 expressing C57Bl/6J lungs may account for the lethality phenotype observed on that background.

**Fig. 4:**
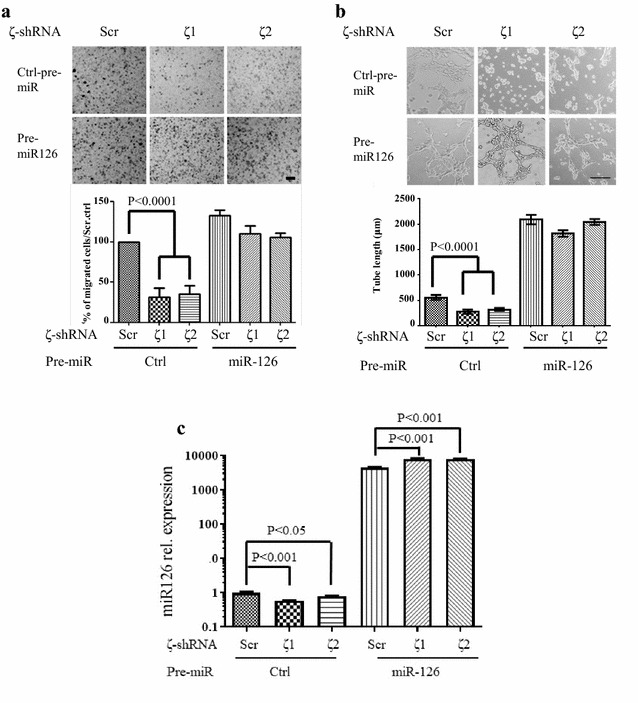
14-3-3ζ and miR-126 regulate endothelial migration and tube formation. **a** Transwell migration assay of mouse endothelial cells with 14-3-3ζ knockdown and rescue by overexpression of exogenous miR-126. Cells stably expressing scramble or 14-3-3ζ shRNA were transfected with control or pre-miR-126. Representative result is shown in the top panel and the bottom panel shows the quantification. Scale bar indicates 50 μm in length. **b** Tube formation assay with exogenously expressed miR-126 in the 14-3-3ζ knockdown mouse endothelial cells. Representative results are shown in the top panel and the bottom panel shows the quantification. Scale bar indicates 50 μm in length. **c** Mouse endothelial cells stably transfected with either scrambled shRNA or shRNA targeting 14-3-3ζ were transfected with pre-miR-126. MiR-126 expression was analyzed by qRT-PCR and normalized to PECAM1, ± SD (n = 3). Three independent transwell experiments were performed and three random fields from the tube formation assay were analyzed for statistical analysis

To determine the contribution of miR-126 downregulation in 14-3-3ζ-loss induced vascular defects, mouse endothelial cells were stably transfected with two distinct shRNAs targeting 14-3-3ζ and a scrambled shRNA was used as a control (Fig. 3c). Loss of 14-3-3ζ expression led to a significant downregulation of miR-126 (Fig. 3d) and inhibited the angiogenic functionality of the endothelial cells as determined by both migration and tube formation assays (Fig. 4a, b). Expression of a miR-126 mimetic (pre-miR-126) (Fig. 4c) in 14-3-3ζ knockdown mouse endothelial cells rescued their migration and tube formation capabilities (Fig. 4a, b lower panels). These results demonstrated that downregulation of the angiogenesis regulator miR-126 plays a critical role in 14-3-3ζ-loss induced lung vasculature defects on the C57Bl/6J background.

**Fig. 5:**
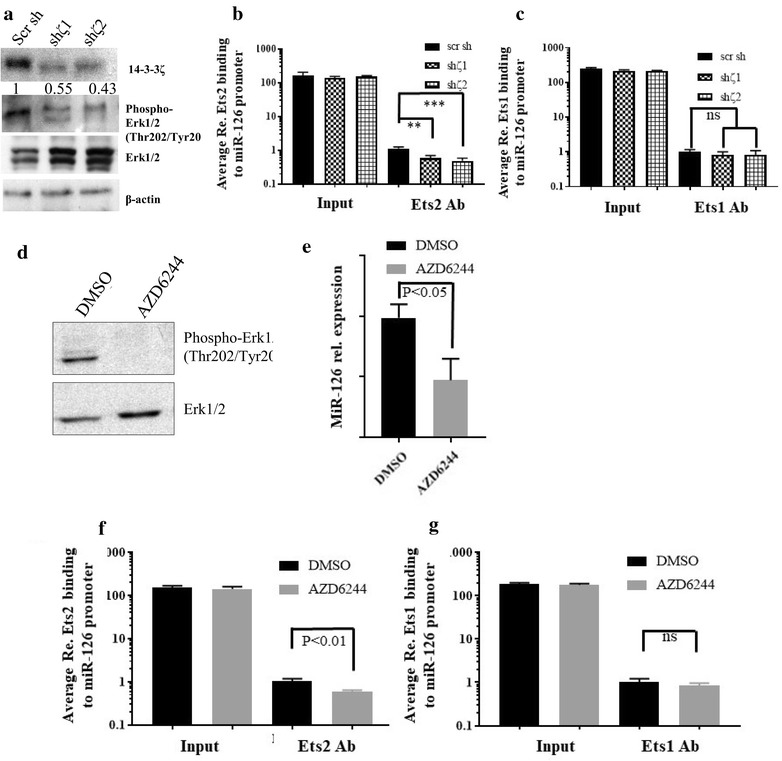
14-3-3ζ mediates miR-126 transcription by modulating Ets2 activity. **a** Detection of 14-3-3ζ, phosphor-Erk 1/2 (Thr202/Tyr204), total Erk1/2 and β-actin by western blotting in mouse endothelial cells expressing either a scrambled shRNA or shRNA targeting 14-3-3ζ. **b**, **c** ChIP analysis of Ets2 and Ets1 binding to the miR-126 promoter in mouse endothelial cells expressing either a scrambled shRNA or shRNA targeting 14-3-3ζ, ± SD (n = 3). **d** Analysis of phosho-Erk (Thr202/Tyr204) and Erk1/2 in mouse endothelial cells treated with either DMSO or the ERK1/2 inhibitor AZD6244 by western blotting. **e** qRT-PCR analysis of miR-126 expression relative to PECAM1 in mouse endothelial cells treated with either DMSO or AZD6244, ± SD (n = 3). **f** ChIP analysis of Ets2 binding to the miR-126 promoter in mouse endothelial cells treated with either DMSO or AZD6244. ± SD. **g** ChIP analysis of Ets1 binding to the miR-126 promoter in mouse endothelial cells treated with either DMSO or AZD6244. ± SD (n = 3)

The Raf/Mek/Erk regulated transcription factors Ets1 and Ets2 are important transcriptional regulators of miR-126 [35]. As 14-3-3ζ plays an important role in Raf-1 activation [36, 37], loss of 14-3-3ζ may result in attenuated activation of the Raf/Mek/Erk pathway leading to decreased Ets1 and Ets2 mediated transcription of miR-126. Indeed, loss of 14-3-3ζ in mouse endothelial cells reduced Erk phosphorylation (Fig. 5a), and attenuated binding of Ets2 to the miR-126 promoter (Fig. 5b) while Ets1 binding was not significantly altered (Fig. 5c). Inhibition of Erk activity in mouse endothelial cells using a MEK1/2 inhibitor (AZD6244) also attenuated miR-126 expression (Fig. 5d, e) and reduced Ets2 binding to the miR-126 promoter (Fig. 5f) while Ets1 binding was unaffected (Fig. 5g). These results suggest that loss of 14-3-3ζ attenuates miR-126 transcription by decreased binding of Ets2 to the miR-126 promoter.

## Discussion

14-3-3 isoforms exhibit unique non-overlapping biological functions. 14-3-3σ stabilizes p53, thus suppresses tumor growth [38–40] and is frequently lost in cancer [41–43]. 14-3-3σ mediates keratinocyte proliferation and differentiation in vivo [44]. 14-3-3ε can enhance TGF-β signaling [45], bind to poly A polymerase and regulate its cellular localization [46]. Targeted disruption of 14-3-3ε in mice results in embryonic lethality from hippocampal and cortical defects while 14-3-3ε heterozygous mice exhibited neuro-developmental defects that mimic the Miller–Dieker syndrome [47]. 14-3-3γ knockout mice do not display a discernible phenotype [48] but it can affect PDGF signaling in smooth muscle cells [49]. 14-3-3τ regulates E2F stability, is required for autophagy [50] and targeted deletion of 14-3-3τ in mice leads to embryonic lethality from gross developmental defects [51]. We uncovered an unexpected role for 14-3-3ζ in mediating vascular integrity during lung development. Thus loss of 14-3-3ζ in mice leads to distinct physiological defects and pathological effects in various genetic backgrounds that cannot be compensated by other 14-3-3 isoforms in vivo. Together, these studies indicated that specific 14-3-3 isoforms, including 14-3-3ζ, have distinctive biological functions in normal development and in diseases.

Loss of 14-3-3ζ leads to neonatal lethality with defects in lung development on the C57Bl/6J genetic background, indicating that 14-3-3ζ plays an indispensable role in normal development. Mechanistic insights came from our finding that loss of 14-3-3ζ on the C57Bl/6J background phenocopies miR-126 knockout mice. MiR-126 is an endothelial cell-specific microRNA, and miR-126 loss resulted in neonatal lethality and lung deflation due to vascular disintegration and inhibition of angiogenesis [34]. Additionally, zebrafish studies have suggested a role of miR-126 in facilitating the lung vasculature integrity by reducing the expression of negative regulators of the VEGF pathway, such as SPRED-1 (Sprouty related protein 1) and the PI3K regulatory subunit 2 [52]. Several miR-126 targets have been identified that potentially account for the importance of miR-126 expression in endothelial cells. MiR-126 has also been shown to enhance endothelial cell proliferation by targeting the 3′UTR of the Notch1 inhibitor delta-like 1 homolog (Dlk1) [53]. MiR-126 was also found to suppress the expression of the endothelial adhesion molecule vascular cell adhesion molecule 1 (VCAM1) and thereby plays a role in regulating vascular inflammation [54]. Other miR-126 targets that play a role in angiogenesis include VEGF [55], epidermal growth factor like domain 7 (EGFL7) [56], etc. The lungs of our 14-3-3ζ−/− mice had a significant miR-126 down-regulation, consistent with the defective lung development phenotype. Furthermore, reintroducing miR-126 to 14-3-3ζ knockdown endothelial cells rescued endothelial cell functions. Together, these data indicate that miR-126 down-regulation plays a critical role in the defective lung vasculature-mediated respiratory failure and neonatal lethality in 14-3-3ζ knockout mice, which may also have relevance for clinical syndromes of neonatal respiratory distress [57]. Notably, 14-3-3ζ binds to many target proteins and regulates a wide variety of biological processes, thus miR-126 down-regulation in 14-3-3ζ−/− mice is likely one of the mechanisms underlying the developmental defects. A more comprehensive and in-depth future study will define other molecular mechanisms at play.

**Fig. 6:**
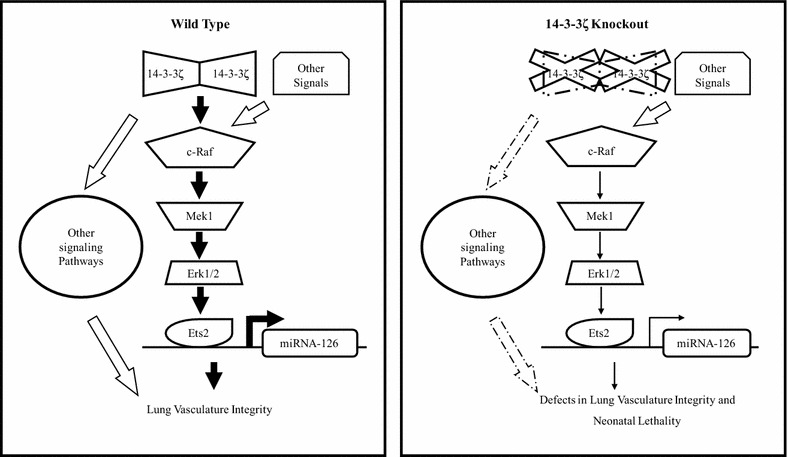
Schematic model of 14-3-3ζ mediated lung vascular integrity regulation and respiratory distress mediated neonatal lethality. On C57Bl/6J background, 14-3-3ζ is critical for the activation of multiple signaling pathways including the Raf/Mek/Erk pathway, facilitating the Ets2 mediated transcription of the pro-angiogenic miR-126, which promotes lung vascular integrity (left). In the absence of 14-3-3ζ, the Raf/Mek/Erk/Ets2 pathway, along with other signaling pathways, is inhibited and miR-126 expression is attenuated, resulting in defects in lung vascular integrity that leads to neonatal lethality (right)

14-3-3s have been previously implicated to regulate angiogenesis in vitro and in lower organisms [58]. In our 14-3-3ζ−/− mouse model, loss of 14-3-3ζ in endothelial cells clearly inhibited angiogenesis during development in vivo and miR-126 downregulation contributes to this phenotype. Since 14-3-3ζ plays an important role in activation of Raf/Mek/Erk pathway [36, 59], loss of 14-3-3ζ leads to inhibition of the Raf/Mek/Erk pathway. Erk downstream targets, Ets1 and Ets2 [60], are important regulators of miR-126 transcription [35]. Indeed, loss of 14-3-3ζ in mouse endothelial cells resulted in reduced Erk activity, attenuated binding of Ets2 to the miR-126 promoter, and ultimately repressed miR-126 expression (Fig. 5). Mice lacking both Ets1 and Ets2 have been reported to be embryonic lethal due to vascular defects and enhanced endothelial cell apoptosis [61], indicating an important role for Ets1 and Ets2, the miR-126 regulators, in maintenance of vascular integrity. Ets1 and Ets2 were found to regulate the expression of angiogenic genes such as matrix metalloprotease 9 (MMP9), Bcl-X(L) and c-IAP2 [61]. Our findings that loss of 14-3-3ζ led to reduced Ets2 binding to the miR-126 promoter resulting in miR-126 downregulation and inhibited angiogenesis in 14-3-3ζ−/− mouse model clearly established 14-3-3ζ as an upstream regulator of the Erk/Ets2/miR126/angiogenesis axis (Fig. 6).

Previously, we reported that 14-3-3ζ regulates transcription of miR-221 via the Erk target c-fos and the Jnk target c-Jun [62]. In this study, we found that 14-3-3ζ regulates miR-126 via the Erk/Ets2 pathway. These suggest an important role for 14-3-3ζ in controlling miRNA transcription via its regulation of the Raf/Mek/Erk pathway. Ets1 and Ets2 have been shown to regulate the transcription of multiple miRNAs such as miR-196b [63], miR-155 [64] which also play an important role in mediating endothelial cell function [65, 66]. Additionally Ets2 regulates the transcription of c-Myc [67] which can regulate miRNA processing via regulating drosha transcription [68]. Thus, it is plausible that 14-3-3ζ may regulate miRNA transcription and miRNA processing, ultimately miRNA expression and activity, which merits future in-depth studies. Additional tissue/organ defects due to 14-3-3ζ loss and other mechanisms may also contribute to the neonatal lethality observed on the C57Bl/6J genetic background.

The lethality phenotype from 14-3-3ζ loss was most severe on a C57Bl/6J genetic background and could be rescued by outbreeding to CD-1 and backcrossing to FVB/NJ, suggesting that the genetic background plays an important role in this phenotype. The molecular mechanisms of this dramatic phenotypic difference in different genetic backgrounds remains to be fully understood. Since 14-3-3ζ can bind to many different target proteins to regulate various biological processes, it is possible that one or more such target proteins important for lung development are coded by gene(s) hypomorphic on the C57Bl/6J genetic background. Interestingly, we have observed that miR-126 expression in the lungs of C57Bl/6J mice is significantly lower as compared to that in the FVB/NJ and CD-1 mice, suggesting that downregulation of miR-126 from 14-3-3ζ-loss may lead to more severe biological consequences on the C57Bl/6J mice than in the FVB/NJ and CD-1 mice. Identifying the genes differentially expressed on the C57Bl/6J mouse strain that contributed to 14-3-3ζ-mediated lethality may have important clinical implications. For example, the genes that contributed to 14-3-3ζ-mediated lethality on the C57Bl/6J mouse strain may predict patients’ therapeutic response versus adverse side effects when targeting the 14-3-3ζ and downstream pathways which can guide the optimization of personalized therapy in the future.

## Conclusions

14-3-3ζ plays important functions in mouse lung development. Loss of 14-3-3ζ resulted in neonatal lethality in C57Bl/6 genetic background due to respiratory distress. 14-3-3ζ hypomorphic lungs had dysregulated angiogenesis mediated by inhibition of Erk-Ets2-miR-126 signaling pathway.

## Additional file

**Additional file 1: Figure S1.** Characterization of the ES cell line RRR334. **A** RT-PCR to confirm that the cell line traps 14-3-3ζ schematic view of the integration of the gene trap vector in the 14-3-3ζ gene as described in the legend of Fig. 1. The arrowheads indicate the primers for PCR. Endogenous 14-3-3ζ is expressed both in wild-type ES cell control, TC1, and the mutant cell line RRR334. The exogenous mutant allele exists only in the RRR334 cell line. **B** Determination of the integration site of the gene trap vector using PCR. Arrowheads indicate the primers for PCR. The numbers on each lane of the gel indicate the primer position in the 14-3-3ζ gene. “N” indicates negative control; “M” indicates marker. The 1636 and 506-bp marker sizes are shown. **C** Western blot analysis of 14-3-3ζ expression level in 8 week old female B6/129 mice mammary gland. Quantification of relative 14-3-3ζ expression level is shown below the 14-3-3ζ blot panel. **Figure S2.** Characterization of truncated 14-3-3ζ. **A** Western blot of lysate of MCF7 vector control transfectants (Vc) and two MCF7 transfectants of HA-tagged N-terminal fragment 139 amino acids of 14-3-3ζ [C-terminal deletion (ΔC1 and ΔC12)]. Cells were treated with DMSO or 50 nM MG132 for 4 h. Endogenous 14-3-3ζ was detected using 14-3-3ζ antibody, while the exogenous 14-3-3ζ C-terminal deletion fragment was detected using HA antibody. **B** 14-3-3ζ N-terminal fragment did not affect p-Mek1 and p-Akt levels. Western blot on lysates from the indicated transfectants were performed with indicated antibodies. β-Actin was used as loading control. **C** 14-3-3ζ N-terminal fragment did not affect proliferation in MCF-7 cells. MTT assay was performed on the three indicated transfectants. OD was measured at 570 nm and normalized to 650 nm. **Figure S3.** 14-3-3ζ expression in FVB/NJ and CD-1 14-3-3ζ+/+ and 14-3-3ζ−/− mice. **A** Analysis of 14-3-3ζ and β-actin in different organs in the CD-1 14-3-3ζ+/+ and 14-3-3ζ−/− mice by western blotting. Quantification of relative 14-3-3ζ expression level is shown below the western panel. **B** Analysis of 14-3-3ζ and β-actin in different organs in the FVB/NJ 14-3-3ζ+/+ and 14-3-3ζ−/− mice by western blotting. Quantification of relative 14-3-3ζ expression level is shown below the western panel. **C** Analysis of 14-3-3ζ, 14-3-3β, 14-3-3ε and β-actin in the liver, kidney and lungs from FVB/NJ 14-3-3ζ+/+ and 14-3-3ζ−/− mice by western blotting. Quantification of relative 14-3-3ζ, 14-3-3β, 14-3-3ε expression level is shown below the western panel.
