# Supplementary material for: 14-3-3ζ loss leads to neonatal lethality by microRNA-126 downregulation-mediated developmental defects in lung vasculature
Source: Cell Biosci. 2017 Nov 2;7:58. doi: 10.1186/s13578-017-0186-y (PMC5667492; doi:10.1186/s13578-017-0186-y)
Supplement: Supplementary file 1 — Additional file 1: Figure S1. Characterization of the ES cell line RRR334. A RT-PCR to confirm that the cell line traps 14-3-3ζ schematic view of the integration of the gene trap vector in the 14-3-3ζ gene as described in the legend of Fig. 1. The arrowheads indicate the primers for PCR. Endogenous 14-3-3ζ is expressed both in wild-type ES cell control, TC1, and the mutant cell line RRR334. The exogenous mutant allele exists only in the RRR334 cell line. B Determination of the integration site of the gene trap vector using PCR. Arrowheads indicate the primers for PCR. The numbers on each lane of the gel indicate the primer position in the 14-3-3ζ gene. “N” indicates negative control; “M” indicates marker. The 1636 and 506-bp marker sizes are shown. C Western blot analysis of 14-3-3ζ expression level in 8 week old female B6/129 mice mammary gland. Quantification of relative 14-3-3ζ expression level is shown below the 14-3-3ζ blot panel. Figure S2. Characterization of truncated 14-3-3ζ. A Western blot of lysate of MCF7 vector control transfectants (Vc) and two MCF7 transfectants of HA-tagged N-terminal fragment 139 amino acids of 14-3-3ζ [C-terminal deletion (ΔC1 and ΔC12)]. Cells were treated with DMSO or 50 nM MG132 for 4 h. Endogenous 14-3-3ζ was detected using 14-3-3ζ antibody, while the exogenous 14-3-3ζ C-terminal deletion fragment was detected using HA antibody. B 14-3-3ζ N-terminal fragment did not affect p-Mek1 and p-Akt levels. Western blot on lysates from the indicated transfectants were performed with indicated antibodies. β-Actin was used as loading control. C 14-3-3ζ N-terminal fragment did not affect proliferation in MCF-7 cells. MTT assay was performed on the three indicated transfectants. OD was measured at 570 nm and normalized to 650 nm. Figure S3. 14-3-3ζ expression in FVB/NJ and CD-1 14-3-3ζ+/+ and 14-3-3ζ−/− mice. A Analysis of 14-3-3ζ and β-actin in different organs in the CD-1 14-3-3ζ+/+ and 14-3-3ζ−/− mice by western blotting. Quantification o [file 13578_2017_186_MOESM1_ESM.pptx]

## Slide 1
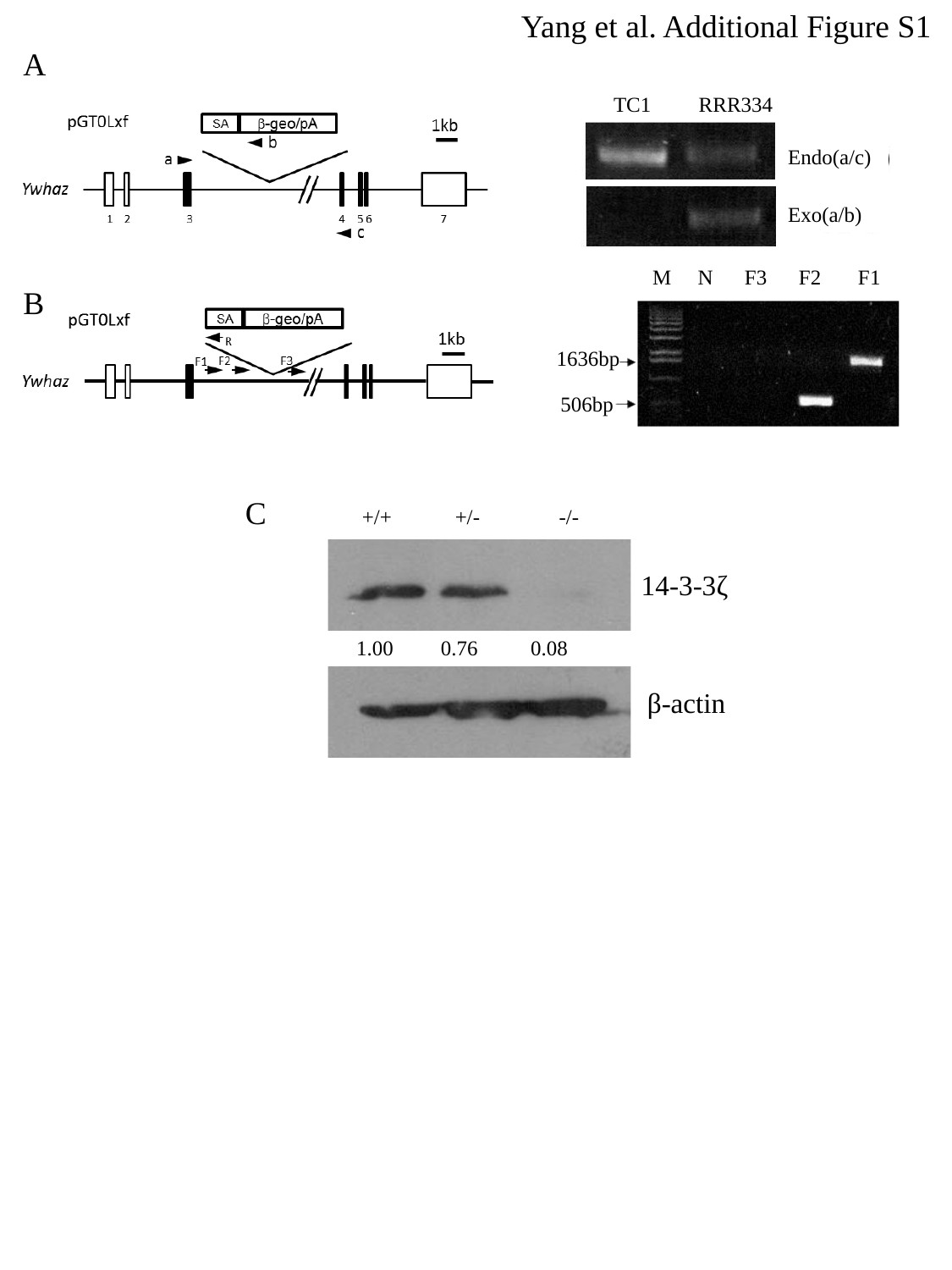

Yang et al. Additional Figure S1
A
 TC1 RRR334
 Endo(a/c)
 Exo(a/b)
 M N F3 F2 F1
B
1636bp
506bp
C
+/+ +/- -/-
14-3-3ζ
 1.00 0.76 0.08
β-actin

## Slide 2
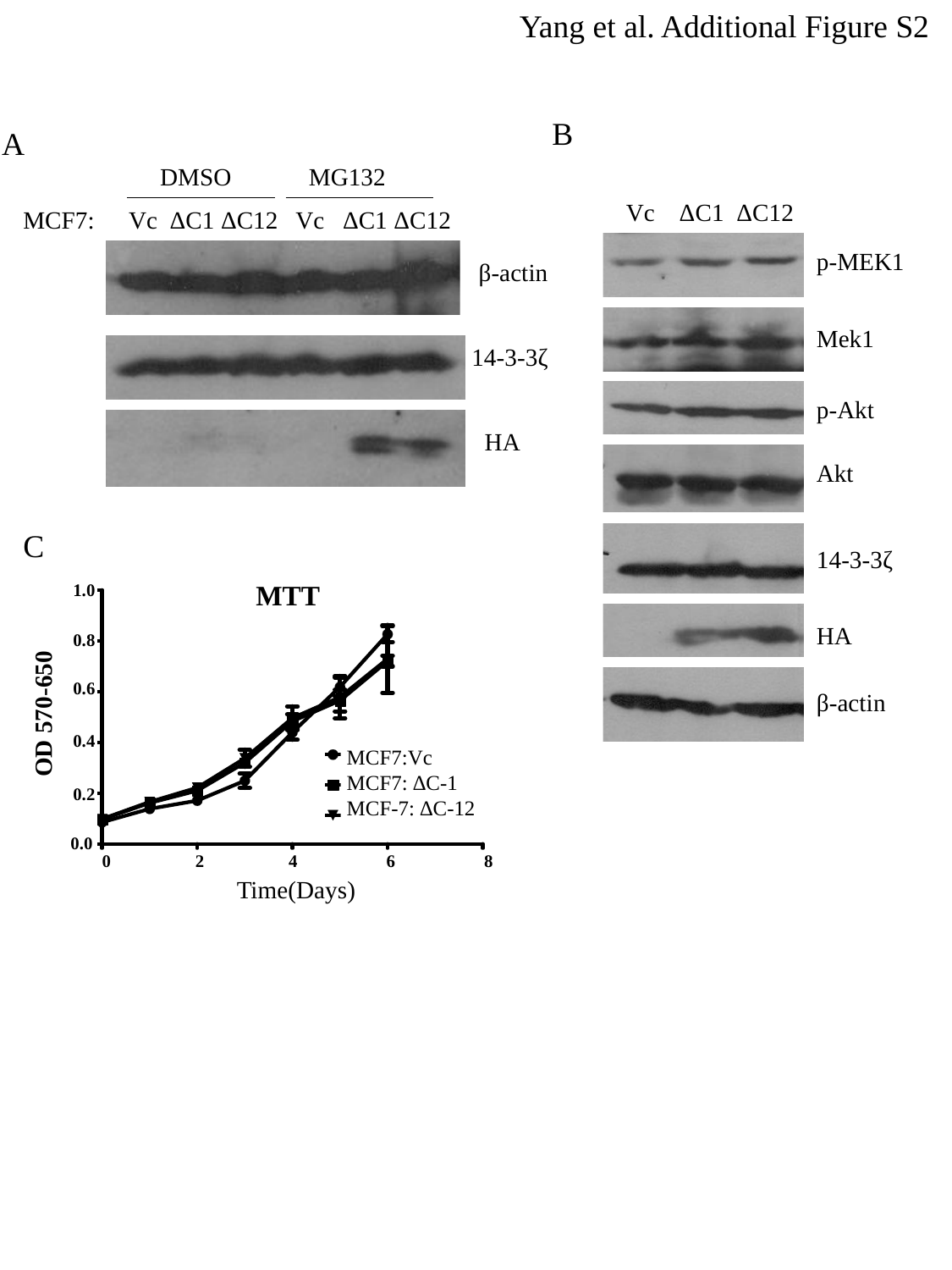

Yang et al. Additional Figure S2
B
A
DMSO
MG132
Vc ΔC1 ΔC12
Vc ΔC1 ΔC12
MCF7:
β-actin
14-3-3ζ
HA
Vc ΔC1 ΔC12
p-MEK1
Mek1
p-Akt
Akt
14-3-3ζ
HA
β-actin
C
MTT
1.0
0.8
0.6
OD 570-650
0.4
MCF7:Vc
MCF7: ∆C-1
MCF-7: ∆C-12
0.2
0.0
 0 2 4 6 8
Time(Days)

## Slide 3
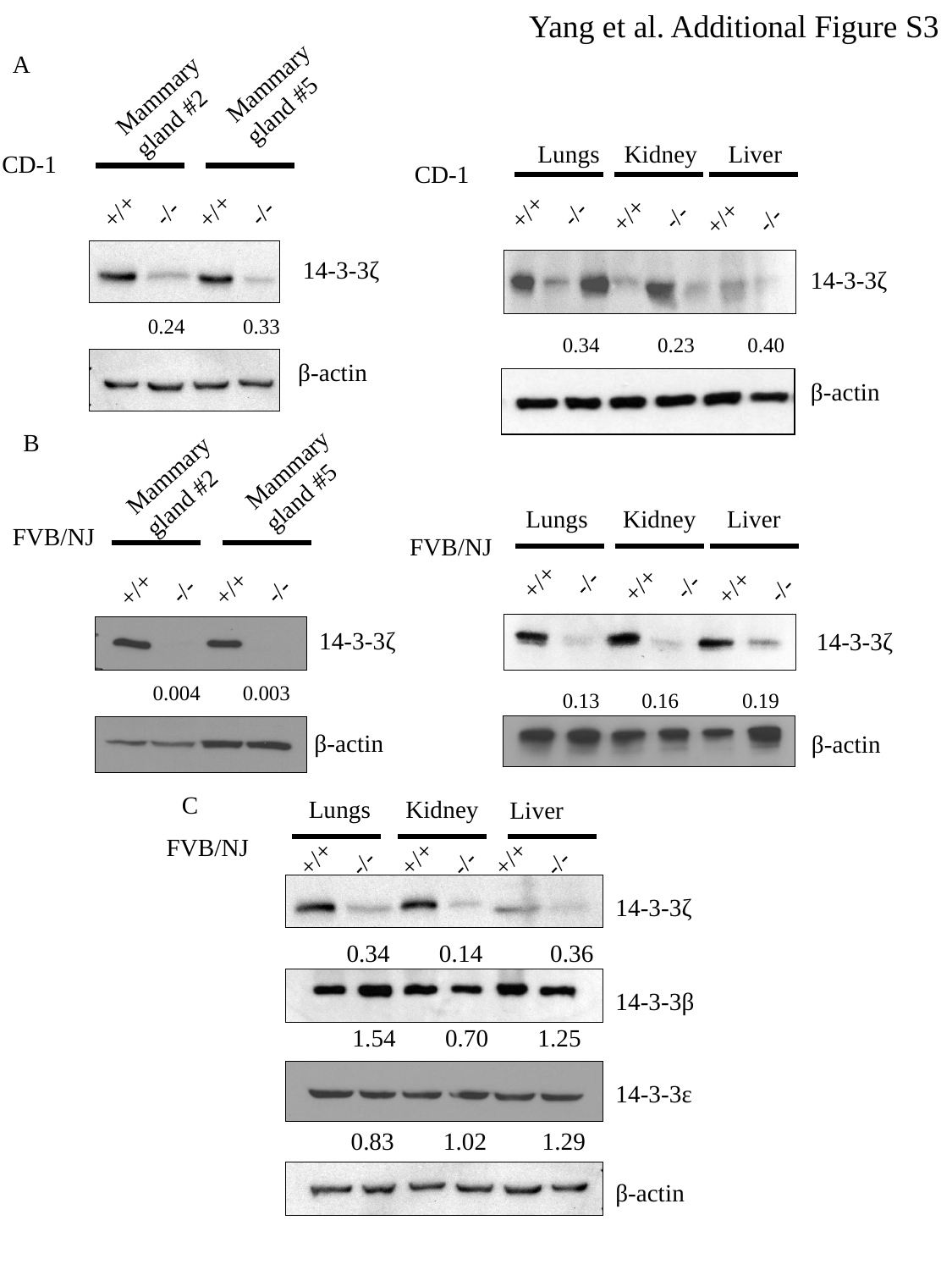

Yang et al. Additional Figure S3
-/-
+/+
-/-
+/+
-/-
+/+
Mammary gland #5
A
Mammary gland #2
-/-
-/-
+/+
+/+
Kidney
Lungs
Liver
CD-1
CD-1
14-3-3ζ
14-3-3ζ
 0.24 0.33
 0.34 0.23 0.40
β-actin
β-actin
-/-
+/+
-/-
+/+
-/-
+/+
B
Mammary gland #5
Mammary gland #2
-/-
-/-
Lungs
Kidney
+/+
Liver
FVB/NJ
FVB/NJ
+/+
14-3-3ζ
14-3-3ζ
 0.004 0.003
 0.13 0.16 0.19
β-actin
β-actin
+/+
-/-
+/+
-/-
+/+
-/-
C
Lungs
Kidney
Liver
FVB/NJ
14-3-3ζ
 0.34 0.14 0.36
14-3-3β
 1.54 0.70 1.25
14-3-3ε
 0.83 1.02 1.29
β-actin
